# NADPH Oxidases in Aortic Aneurysms

**DOI:** 10.3390/antiox11091830

**Published:** 2022-09-16

**Authors:** Morgan Salmon

**Affiliations:** Department of Cardiac Surgery, Michigan Medicine, University of Michigan, Ann Arbor, MI 48109, USA; msalmon@med.umich.edu

**Keywords:** aortic aneurysm, reactive oxygen species, NADPH oxidases/NOX

## Abstract

Abdominal aortic aneurysms (AAAs) are a progressive dilation of the infrarenal aorta and are characterized by inflammatory cell infiltration, smooth muscle cell migration and proliferation, and degradation of the extracellular matrix. Oxidative stress and the production of reactive oxygen species (ROS) have been shown to play roles in inflammatory cell infiltration, and smooth muscle cell migration and apoptosis in AAAs. In this review, we discuss the principles of nicotinamide adenine dinucleotide phosphate oxidase (NADPH oxidase/NOX) signaling and activation. We also discuss the effects of some of the major mediators of NOX signaling in AAAs. Separately, we also discuss the influence of genetic or pharmacologic inhibitors of NADPH oxidases on experimental pre-clinical AAAs. Experimental evidence suggests that NADPH oxidases may be a promising future therapeutic target for developing pharmacologic treatment strategies for halting AAA progression or rupture prevention in the management of clinical AAAs.

## 1. Introduction

Aortic aneurysms are defined as a localized dilation of the aorta and can be classified into three sub-types by their location within the aorta: ascending aortic aneurysms (AAs), descending thoracic aortic aneurysms (dTAAs), and abdominal aortic aneurysm (AAAs). While all types are generally clinically silent phenomena until impending rupture, TAAs tend to dilate until impending dissection, with approximately 30% of cases having a genetic component from either (1) a clinical syndrome such as Marfans, Loeys Dietz, or vascular Ehlers–Danlos, or (2) a genetic predisposition such as Bicuspid Aortic Valve, or familial Thoracic Aortic Aneurysm and Dissection [1]. In contrast, AAAs tend to dilate until impending rupture and most cases have no genetic predisposition to formation or progression [2,3,4,5,6]. Often asymptomatic, AAAs can quickly expand and undergo rupture, resulting in 80–90% mortality after rupture and 13,000–15,000 deaths per year in the United States [7,8]. Common to both types of aneurysms is the destruction of the extracellular matrix, an influx of inflammatory cells, the activation of pro-inflammatory cytokines, and apoptosis of smooth muscle cells that leads to eventual dissection or rupture.

Aneurysms tend to display heterogeneity in terms of their clinical severity, and continued progressive dilation of the aorta without intervention will no doubt lead to lethal aortic rupture with high morbidity and mortality rates [7,8]. In terms of AAAs, to date, the most significant predictive factor of aortic rupture is maximal aortic dilation [9,10]. Other factors suggested to affect AAA progression include smoking status, biological sex, rate of growth, and hemodynamics conditions [11,12,13,14,15,16,17,18,19]. Current clinical recommendations suggest surgical intervention using either open or endovascular therapy for AAAs at 55 mm; however, some AAAs rupture at sizes smaller than current recommendations [20]. The lack of known causes of AAA progression or rupture suggest that mechanisms of AAA progression and rupture remain poorly understood. These currently unknown mechanisms complicate the identification of medical treatment therapies specific for AAAs that could halt progression or prevent rupture [2,3,4]. Advances in imaging technology and screening programs have led to increased identification of early stage AAAs [21,22,23,24,25,26,27,28,29]; however, without a medical treatment therapy, these AAAs can only be monitored until clinical recommendations suggest surgical intervention. Recent evidence suggests that, despite these increased screening efforts and attempts at prevention management, AAAs remain high in the United Kingdom for both men and women with age-standardized death rates (ASDR) of 7.5 per 100,000 and 3.7 per 100,000, respectively, and the ASDR in many European countries for AAAs have increased slightly since 2012 despite national screening efforts [30]. These data suggest that additional methods are needed to monitor AAAs and that AAAs remain a relevant health concern despite declines from smoking cessation. In this review, we discuss the role of nicotinamide adenine dinucleotide phosphate (NADPH) oxidases and their known downstream mediators in aortic aneurysm formation and rupture.

## 2. Nicotinamide Adenine Dinucleotide Phosphate Oxidases (NADPH Oxidases/NOX)

Nicotinamide adenine dinucleotide phosphate oxidase (NADPH oxidase/NOX) comprise a multi-subunit enzyme complex that utilizes nicotinamide adenine dinucleotide phosphate to produce both superoxide anions and other reactive oxygen species. Under normal circumstances, reactive oxygen species (ROS) mediate several important cellular functions, including the maintenance of blood pressure and adaptive immunity. However, high levels of ROS over prolonged periods of time can lead to cellular damage, oxidative stress, and DNA damage, which elicit either cell survival or apoptosis mechanisms depending on severity and duration of exposure. Prolonged exposure to ROS is hypothesized to be a hallmark of AAA formation [31,32]. ROS includes free radicals such as superoxide (O^2−^), hydroxyl radical (OH), and non-radicals such as hydrogen peroxide (H_2_O_2_). Increased endothelial permeability has been linked to ROS activation and is believed to play a critical role in initiation of vascular diseases such as atherosclerosis and aortic aneurysms. The effects of ROS on the endothelium are discussed in greater detail in Section 3 of this review.

**Figure 1:**
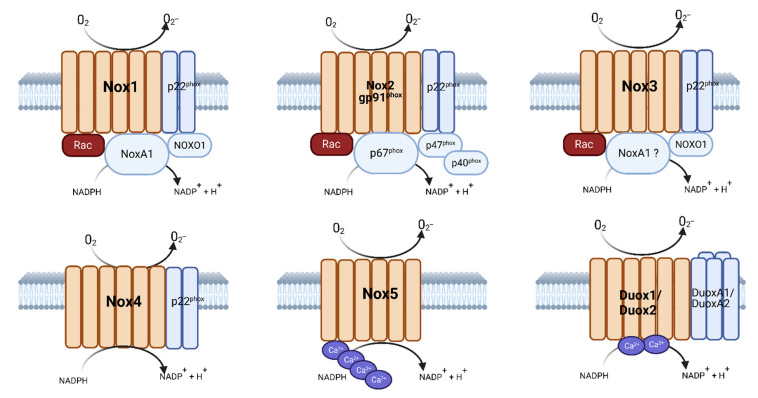
**Structure of the Nox family members’ complexes.** Created using Biorender.com and adapted from [50].

The main function of NADPH oxidases/NOX, a family of enzymes implicated in cardiovascular diseases, is to produce ROS [33]. The first characterized NADPH oxidase was defined in neutrophils and macrophages and is known to be a multi-component complex that catalyzes the formation of O_2_^•−^ during phagocytosis [34]. In the resting cell, the NADPH oxidase contains a membrane-bound catalytic core of the enzyme, flavocytochrome b_55_8, and cytosolic regulatory subunits p47phox, p40phox, p67phox, and small G-protein Rac1 or Rac2 (Figure 1). The flavo-cytochrome b_55_8 is the catalytic center of NOX and comprises two tightly complexed membrane-integrated flavocytochromes: gp91-phox and p22-phox [35]. Meanwhile in the cytosol, the cytosolic components of the complex contain p47-phox, p67-phox, and p40-phox and the small GTPase Rac1/Rac2, with the p40-phox and p67-phox proteins often being complexed prior to activation [36,37,38]. In resting phagocytic cells such as macrophages, p47phox, p67phox, and p40phox are demonstrated to exist as complexes in the cytosol that are stabilized by SH3 domain interactions [39]. Conversely, Rac is tethered to RhoGDI, a RhoGDP-dissociation inhibitor, that prevents its binding to the rest of the NOX complex [40]. In the resting state, binding to the flavocytochrome is prevented because p47phox exists in an auto-inhibited conformation, a positional conformation that prevents its tandem SH3 domains from being exposed, and they are masked through intramolecular interaction with the C-terminal segment, preventing activation of the complex.

During NOX activation in the phagocytic cell, phosphorylation unmasks a binding region on p47-phox, allowing it to bind p67-phox to form a trimeric cytosolic complex [39,41]. The phosphorylation sites consist of multiple serine residues in the C-terminus of p47phox, changing the physical conformation of p47-phox to enable the N-terminal SH3 domain to allow for eventual interaction with the proline-rich region of p22phox at the membrane [42,43,44,45]. Following the formation of the trimeric cytosolic complex, p47-phox mediates translocation of the cytosolic complex to the membrane, where it then binds to p22-phox; the active NOX complex is assembled; and activation of gp91-phox can then occur [46]. Since gp91-phox is the catalytic core, gp91-phox levels are an established measurement for the extent of NOX complex formation. The gp91-phox NOX protein family contains NADPH- (or NADH-) binding domains, which use NADPH as electron donors to produce superoxide anions (O2^•–^, the precursor for other reactive oxygen species) [36,47]. Thus, glucose metabolism and the electron transport chain provide the NADPH necessary for NOX full function [48,49].

The NOX family consists of several catalytic isoforms and includes seven members: NOX 1–5, Duox1, and Duox2 [35,40]. The structure of NOXes 1–4 are related and contain an N-terminal transmembrane region with six α-helical domains with four conserved histidines. These histidine amino acids are conserved, and two each are located in the third and fifth α-helical domains spanning two asymmetrical hemes. The cytoplasmic C-terminus dehydrogenase domains are also similar and contain conserved binding sites for FAD and NADPH. NOX5 is distinct and contains a calmodulin-like EF domain with four Ca^2+^-binding sites in the long N-terminus. The EF domain in Nox5 allows this enzyme to be sensitive to elevated cytosolic Ca^2+^ levels and can quickly activate in response to cytosolic Ca^2+^ levels [51,52]. Finally, the DUOX proteins are the most divergent members of the NOX family as they are characterized by an N-terminal peroxidase-like domain connected to the EF domain by an additional transmembrane domain [53,54].

Evidence suggests that the catalytic subunits of the NOX family members function distinctly despite all being involved in ROS production. NOX1 and NOX2 function by generating O2^•−^ through the transfer of two electrons from NADPH in the cytosol to FAD and then to the two heme groups via the electron transport chain [35]. NOX4 generates H_2_O_2_, through two conserved cysteines, Cys226 and Cys270, and a highly conserved His222 residue in the third extracytosolic loop [55]. It is postulated that the histidine serves as a source of protons for the spontaneous dismutation of O2^•−^ forming H_2_O_2_ [56]. ROS production from this family can be extracellular or intracellular depending on the biological membrane where the NOX family member is located. Activated NOX family members have been found on the plasma membrane, endosome, phagosome, caveolae, endoplasmic reticulum, mitochondria, and nucleus [57].

NOX family members have been shown to be expressed across various cell types in the vascular system, and over-activation has been suggested to be critical for diseases such as atherosclerosis and aortic aneurysms [58]. NOX1 is expressed in smooth muscle cells (SMCs) [48,57,59,60], endothelial cells [59,61], and fibroblasts [59]. NOX2 is expressed in endothelial cells [46,59,62,63,64], fibroblasts [65], cardiomyocytes [66,67], and SMCs [68]. NOX4 is expressed in SMCs [59,69,70,71], endothelial cells [46,59], fibroblasts [59,72], and cardiomyocytes [73,74]. NOX5 has been shown to be present in SMCs [75] and endothelial cells [76]. Interestingly, NOX3 and DUOX2 expression have not been reported in vascular cells, while DUOX1 has been reported in SMCs [77]. These data suggest that NOX family member expression varies across different vascular cell types and could play distinct roles in vascular disease mechanisms.

## 3. Mediators of NADPH Oxidases/NOX in Experimental AAAs

The NOX Family is known to exert effects on down-stream mediators in multiple cell types in vascular diseases. We specifically discuss the effects of several major mediators of NOX signaling within the context of experimental AAAs [78].

### 3.1. Reactive Oxygen Species (ROS)

Inflammatory vascular diseases, such as atherosclerosis and AAAs, are believed to be strongly linked to the over-production of ROS. An important initiating event in the inflammatory vascular disease process is the disruption of flow in the vasculature leading to increased ROS production and activation of endothelial cells. The overproduction of ROS may subsequently induce inflammation, matrix metalloproteinase (MMP) activity, smooth muscle cell apoptosis, or changes in collagen properties [79]. Studies have found that NADPH oxidases and iNOS are producers of superoxide (O_2_^•−^) anions in AAAs [79,80] (Table 1). These studies demonstrated that superoxide anions are increased in AAAs in humans and that this increase could be linked to increased NADPH oxidase and iNOS production in AAAs. Studies in experimental AAAs also demonstrate that NADPH oxidases and iNOS are critical for free radical production in aneurysms [81]. A loss of these enzymes was demonstrated to prevent the development of AAAs through reduced expressions of MMP-2 and MMP-9 in the aortic tissues. Finally, as part of the same study, mice were treated with the oxidase inhibitor apocynin and were shown to be protected from AAA formation [81].

Separate studies in humans have also verified elevated levels of superoxide and lipid peroxidation products in human AAAs and linked these elevated levels to NADPH oxidase activity in AAAs [31]. The inducible form of nitric oxide synthase (iNOS), a source of reactive oxygen species (ROS) and reactive nitrogen species, is upregulated in human AAAs [82], while patients also demonstrated decreased catalase activity, an enzyme known to degrade hydrogen peroxides in vivo [83]. Catalase was shown to be decreased in the aortic wall in human AAAs, and treatment of experimental models of AAAs with catalase was able to attenuate AAA formation [84,85]. These studies demonstrate that ROS are elevated in AAA progression and that the inhibition of ROSs with oxidase inhibitors can attenuate disease formation in pre-clinical experimental models of AAAs.

### 3.2. eNOS

Endothelial nitric oxide synthase (eNOS) protects vascular cells from oxidative damage via the production of nitric oxide (NO^•^) to rapidly inactivate superoxide (O2^•−^) and other reactive oxygen species (ROS). Evidence has demonstrated that, when eNOS cofactor tetrahydrobiopterin (H4B) is deficient, eNOS becomes dysfunctional and begins to produce O2^•−^ rather than NO^•^ [86]. This uncoupling of eNOS is thought to contribute to endothelial dysfunction in vascular disease progression. Interestingly, the aneurysm infusion induction agent, angiotensin II, has been shown to partially function via the uncoupling of eNOS [86]. Gao et al. demonstrated that Ang II uncouples eNOS via transient activation of NADPH oxidase (NOX) and is consequently hydrogen peroxide-dependent, with endothelium-specific deficiency in H4B salvage enzyme dihydrofolate reductase (DHFR) [86]. Folic acid treatment was shown to ameliorate the effects of the uncoupling of eNOS in experimental AAAs [87]. Recent studies have also attempted to link changes in eNOS expression to aged AAAs in humans and mouse and found that eNOS levels decline in aged AAAs and could be linked to increased aortic diameters in aging [88].

ENOS has been shown to be activated by TGF-β signaling in vascular diseases such as atherosclerosis or aortic aneurysms [89]. In Marfan syndrome-associated ascending aortic aneurysms, a key molecule, transforming growth factor-beta (TGF-beta), normally bound to the extracellular matrix, is free, activated, and allowed to activate eNOS unchecked. In an experimental setting, TGF-beta blockade prevents the aortic root structural damage and dilatation of ascending aortic aneurysm formation [90]. The Angiotensin receptor 1 blockers (also known as sartanics) exert an anti-TGF-beta effect; trials are now ongoing for evaluating the effect of Losartan compared with atenolol in Marfan syndrome [90,91,92]. A current clinical trial is underway to examine the effects of sartanics or Angiotensin II receptor blockers in Marfan syndrome-associated aortic dissection.

**Table 1:** Mediators of Nox signaling in experimental AAAs.

Mediator	Influence on Experimental AAAs	References
O_2_*^•−^*	Increased inflammation, iNOS, Nox	[79,80]
	Increased free radical production	[81]
H_2_O_2_	Decreased catalase production	[84,85]
eNOS	Uncoupling increases AAAs	[86,87]
	Levels decline in aged AAAs	[88]
HMGB1	Increased and correlated with MMP2 and MMP9	[93]
	Decreased aneurysm, decreased elastin breakage	[94]
HIF-1α	Elevated in aneurysms	[95]
	Elevated at the site of AAA rupture	[96]
	Stabilized by DFO, increases MMP2	[97]
NF-Κβ	Elevated and pharmacologic inhibition decreased AAAs	[98]
	Endothelial inhibition attenuated AAA formation	[99]

### 3.3. HMGB1

High-mobility group box 1 (HMGB1) is a widely expressed protein that acts as an extracellular signal upon active secretion by immune cells or passive release by dead, dying, and injured cells. HMGB1 plays pivotal roles through both intracellular and extracellular regulation of the cellular response to stress. Although the mechanisms contributing to HMGB1 biology in AAAs are still under investigation, it appears that oxidative stress is a central regulator of HMGB1’s ability to translocate, release, and activate inflammation and cell death in AAAs. High-mobility group box 1 protein (HMGB1) has been shown in several studies to be elevated in AAAs in humans [100], and its genetic inhibition has resulted in attenuated experimental AAAs [93,94]. In the first of these studies by Kohno et al., HMGB1 was elevated in AAAs in humans and positively correlated with matrix metalloproteinase 2 and 9 (MMP2 and MMP9, respectively) expression. Following inhibition using a neutralizing antibody against HMGB1 in experimental AAAs, the expression of MMP2, MMP9, CD68, and TNF-α declined in AAA formation [93]. In separate studies, HMGB1 expression was found to decrease following the mesenchymal stem cell treatment of elastase-induced experimental AAAs. Sharma et al. then eliminated HMGB1 with a neutralizing antibody and found decreased experimental AAAs and decreased IL-17 production [94]. Finally, the study linked HMGB1 activation to NOX2 by eliminating a single allele of NOX2 to find decreased experimental AAAs; decreased MMP2 and 9 activity; and decreased cytokine production of IL-17, IL-23, and IFN-γ [94]. These studies suggest a correlation between NOX2 and HMGB1 in experimental AAAs and demonstrated that NOX2 was able to mediate its inhibitory effects in part via macrophages in the context of experimental AAAs.

### 3.4. HIF-1α

Hypoxia-inducible factor-1 (HIF-1) is a transcription factor found in mammalian cells under reduced oxygen tension that plays an essential role in cellular and systemic homeostatic responses to hypoxia and has a growing importance in vascular diseases. HIF-1 is a heterodimer composed of a 120-kD HIF-1α subunit complexed with a 91- to 94-kD HIF1β subunit. The HIF-1α accumulates in the cytoplasm under hypoxic conditions and translocates to the nucleus to heterodimerize with HIF-1β, forming an active transcription factor. The HIF-1 complex is believed to be a master regulator of oxidative stress gene regulation and has been implicated in the pathogenesis of atherosclerosis, AAA formation, and pulmonary hypertension [101]. HIF-1 has also been shown to be an essential regulator of angiogenesis and macrophage function [95]. HIF-1α was shown to be elevated in human and experimental AAAs. and HIF-1α over-expression could be found at the rupture edge at human AAA tissues [96]. On the other hand, iron chelation has been shown to stabilize HIF-1α by inhibiting the HIF-1α degradation enzyme prolyl hydroxylase (PHD). Treatment with the HIF-1α inhibitors 2-methoxyestradiol and digoxin demonstrated decreased experimental AAAs, while treatment with the PHD inhibitors, cobalt chloride, and JNJ-42041935 did not attenuate experimental AAAs or MMP expression [95]. Finally, studies further investigating the role of iron chelation and HIF-1 expression in experimental AAAs found that deferoxamine (DFO) stabilized HIF-1α expression and promoted increased activation of MMP2 and MMP9 [97].

### 3.5. NF-Κβ

NF-κB transcription factors regulate the expression of hundreds of genes that are involved in regulating cell growth, differentiation, development, and apoptosis. The mammalian NF-κB proteins consist of five different related family members that bind as homodimers or heterodimers to 10-base pair κB sites. All of these family members have a Rel-homology (RHD) domain essential for DNA binding and dimerization. RelA (also known as p65), RelB, and cRel have C-terminal transcription activation domains (TADs) that serve to positively regulate gene expression. The two other mammalian NF-κB proteins are synthesized as larger p105 and p100 precursor proteins, which have C-terminal ankyrin repeats that inhibit DNA binding until partially processed by proteasome to the smaller p50 and p52 products [102]. All NF-κB proteins are capable of homodimerization or heterodimerization with the other NF-κB proteins with the exception of RelB, which can only form heterodimers. Although there are a few exceptions where NF-κB contributes to cell death, in most cases, the expression of NF-κB target genes typically promotes cellular survival. Therefore, the possible roles of NF-κB in relation to Nox family activation are complex in AAAs and require further investigation as their relative roles in relation to each other currently are unknown.

In the context of AAA formation, NF-κB acts as a cytokine-responsive transcription factor that promotes macrophage MMP expression to promote the destruction of the aorta during AAA progression. Several studies have investigated mechanisms of NF-κβ signaling in AAAs [98,99,103]. The first study found that inhibition of NF-κB and ETS in rats could decrease experimental AAAs [103]. Additional studies found that NF-κB expression was elevated in AAAs and that treatment with pyrrolidine dithiocarbamate (PDTC), a pharmacologic inhibitor of NF-κB resulted in decreased IL-1, IL-6, and MMP 9 in experimental AAAs [98]. The importance of endothelial NF-κB signaling was demonstrated by Saito et al. using transgenic mice expressing dominant-negative IκBα selectively in endothelial cells (E-DNIκB mice) [99]. These mice demonstrated both decreased intimal hyperplasia and decreased experimental AAAs in the endothelial specific transgenic deletion of NF-κB in experimental pre-clinical AAA studies.

While these factors are some of the major mediators of NOX family and ROS signaling in vascular diseases, there are additional factors such as JNK and PKC, and other that future studies could determine major roles of these mediators in AAA formation and rupture. As evidence by the current review, ROSs are complex and their mechanisms as they pertain to AAA rupture remain unclear.

## 4. Influence of Genetic NADPH Oxidases/NOX Deficiency Components on Experimental AAAs

Several studies have demonstrated that NOX1 is important for aortic aneurysm formation using the angiotensin II infusion murine model (Table 2). The first of these studies demonstrated that NOX1-/- mice demonstrated decreased AAA size through a change in tissue inhibitor of matrix metalloproteinase 1 (TIMP1) expression [104]. A second study on the hph1 background examined the effects of NOX1-/-, NOX2-/-, and NOX4-/- in angiotensin II infusion treatment therapies [105]. The study found that all three factors decreased aortic rupture rates, decreased aortic aneurysm size, decreased oxidative stress, and decreased eNOS production [105]. In the case of NOX1-/-, in vitro studies were able to link the downregulation of fibrillin 5 to NOX1 in SMCs in aortic dissection, suggesting a possible mechanism for changes in fibrillin 5 expression during vascular disease progression [106]. Studies using conditional NOX1 mice have been investigated in atherosclerosis and demonstrated that the smooth muscle specific elimination of NOX1 increased migration, proliferation and increased phenotypic switching to a macrophage-like state [107]. However, these studies have yet to be performed using aortic aneurysm murine models

The NOX2 isoform has also been investigated in experimental mouse models in several recent studies. The first study coupled the elimination of NOX2 with the hph1 background followed by ANgiotensin II infusion treatment and found decreased rupture, decreased aortic aneurysm size, and decreased eNOS production in the NOX2-/- experimental aneurysm model [105]. The second study investigated the role of NOX2 in the context of NADPH oxidase-dependent high-mobility group box 1 (HMGB1) expression and found that the elimination of a single allele of NOX2 decreased experimental AAAs; decreased MMP2 and 9 activity; and decreased the cytokine production of IL-17, IL-23, and IFN-γ [94]. Interestingly, in the reverse experiments, the over-expression of NOX2 in the endothelium followed by treatment with Angiotensin II did not result in increased aneurysm size or severity despite increased superoxide formation [114]. NOX2 conditional mice have been created and used to study the effects of myeloid specific elimination of NOX2 followed by high fat diet feeding for 16 weeks [115]. Following high fat diet feeding, NOX2-myeloid-specific conditional knock-out demonstrated lower body weight, delayed adiposity, attenuated visceral inflammation, and decreased macrophage infiltration and cell injury in visceral adipose relative to control mice [115]. In addition, the effects of a high fat diet on glucose regulation and circulating lipids were attenuated in the myeloid specific NOX2 conditional knock-out mice. However, no current published studies have investigated NOX2 conditional knock-out mice studies in aortic aneurysms formation.

There have been several studies investigating possible mechanisms of NOX4 in aortic aneurysm formation. The first study, as previously mentioned above, combined elimination of NOX4 on the hph1 background and found that the elimination resulted in decreased rupture, decreased aneurysm size, and decreased eNOS production [105]. A second study examined the role of NOX4 in the Fbn1^C1039G/+^ ascending aneurysm murine model [108]. These studies found that NOX4 was elevated in human and murine aortic aneurysm tissue and that the elimination of NOX4 in the fibrillin ascending aneurysm models decreased aneurysm size and preserved the elastic lamina. NOX4 conditional mice have been investigated in the lung epithelium; however, no studies have investigated the role of cell-specific elimination of NOX4 in aortic aneurysm formation [116].

The possible mechanisms of NOX5 in aortic aneurysm formation appear to diverge from some of the other family members. Interestingly, endothelial-specific NOX5 over-expression in the ApoE-/- model was found to have no effect with a Western diet; however, once these animals became diabetic and were no longer insulin-responsive, they had two times the number of aortic aneurysms of their controls [109]. These studies suggest that NOX5 could play a role in diabetic aortic aneurysms; however, additional studies will be required to further examine the mechanism of this effect.

Three of the NOX family members have unknown effects in aortic aneurysm formation. First, NOX3 levels have been suggested to be undetectable by qPCR in human AAA formation from patient samples and the effects of genetic elimination of NOX3 in experimental AAAs in mouse currently remain unknown [80]. Mice homozygous for a NOX3^het−3J^ mutation are characterized by head tilting and lack otoconia in the utricle and the saccule of the ear; therefore, these vestibular effects could prevent aortic aneurysm studies in adult mice [117]. Second, DUOX1 has been suggested to be elevated in aortic aneurysms in several studies during the investigation of NOX family isoforms; however, genetic murine models in experimental AAAs remain to be investigated [110]. DUOX1 mice have been created and investigated in urothelial cells; however, the effects of genetic elimination of DUOX1 in aortic aneurysm formation remain unknown [118]. Finally, the effects of DUOX2 on experimental AAAs is currently unknown. A Duox2^thyd^ mutation has been mapped to chromosome 2 and identified as a T > G base pair change in exon 16 of DUOX2 in mice [119]. These mice have been used to study the effects of Duox2 in the thyroid and in thyroid-related illnesses.

A number of the additional components of the NOX complex have also been investigated in experimental models of AAAs. In the Angiotensin II experimental models, studies in the LDR-/- background with p47^phox^ elimination demonstrated decreased aortic rupture rates, decreased oxidative stress, and decreased MMP2 levels [112]. A second set of studies on the hph1 background with angiotensin II administration found similar decreases in rupture rates, aneurysm size, and eNOS production [105]. Other family members such as p22^phox^ [31,80,111], p22^phox^ [80,111], Rac1 [120], and Rac2 [113] are known to be elevated in thoracic and abdominal aortic aneurysms, but their specific effects via genetic elimination in experimental AAAs in murine models remain to be determined. These data suggest that there exists a knowledge gap in the mechanisms surrounding NADPH oxidase function in aortic aneurysm formation and that there currently exists tools that could help to fill that knowledge gap in the near future.

## 5. Influence of Pharmacologic NADPH Oxidases in Experimental AAAs

There is a growing body of evidence in experimental AAAs to suggest that pharmacologic targeting of the NOX family and ROS could result in attenuated AAAs. First, there have been a series of studies in humans designed to link ROS inhibition with antioxidant consumption [121,122]. These studies suggest that the consumption of vitamins C, E, or β-carotene could reduce ROS produce and, thus, AAA formation. Related to this study, the treatment of murine AAAs with resveratrol, a polyphenol, resulted in decreased AAA formation and elevated phox-p47 levels [123]. In separate studies, Quercetin, a polyphenol and antioxidant, also demonstrated decreased AAA formation and decreased HIF-1α and VEGF signaling in experimental AAAs [124]. These studies using Quercetin also treated with Celecoxib, a known anti-inflammatory, separately as a positive control to confirm the ability to attenuate AAA formation. These studies suggest that antioxidants or consumption of polyphenols could attenuate AAA formation and progression through the alleviation of oxidative stress. Finally, the drug azathioprine was also found to be a direct inhibitor of Rac1 and cJun in endothelial cells and could decrease AAA progression in an Angiotensin II murine model of experimental AAAs [120].

Increased focus in experimental AAAs has been through targeting the inhibition of several of the downstream mediators of ROS activation. PPARα is a known downstream mediator of ROS activation, and the PPARα antagonist Pemafibrate was shown to not decreased AAA size but to decrease rupture rates in an Angiotensin II murine model of AAA formation [125]. Pemafibrate was also shown to decrease ROS production in the Angiotensin II murine model and in human SMCs [125]. Studies have also investigated whether inhibition of HIF-1α, a downstream mediator of ROS activation, could result in attenuated experimental AAAs. Studies found that 2-methoxyestradiol and digoxin, known inhibitors for HIF-1α, could attenuate AAA formation in experimental pharmacologic prevention and small AAAs [95]. These studies suggest that the inhibition of the downstream mediators of ROS signaling could attenuate AAA formation.

Finally, studies are ongoing to investigate NOX family pharmacologic inhibition in other diseases [5,126], and as possible isoform selective inhibitors become more widely available, these targets may also be used as inhibitors to attenuate AAAs. Recent studies have highlighted specific inhibitors for NOX1 (ML171, GKT136901, and GKT137831 [127,128,129]); NOX 2 (GSK2795039 [130], CYR5099 [131], Bridged tetrahydroisoquinolines: CPP11G and CPP11H [132], Perhexiline, and Suramin (cell impermeable) [133,134]); NOX4 (GLX7013114 [135], GKT137831 [129], GKT137928 [136], ACD084 [137], and Rosmarinic acid [138])], and DUOX1 (Acrolein [139]). These studies were in models of other diseases and have not been investigated as possible inhibitors of aortic aneurysm formation via NADPH oxidase inhibition. In the documented cases examined in this review, few investigated pharmacologic treatment of chronic, well-established AAAs such as those seen in humans. Past possible pharmacologic treatment therapies for AAAs with great treatment potential in experimental pre-clinical AAA models, such as doxycycline, failed to translate into clinical treatment therapies [4]. Part of the reason for the failure to develop treatment therapies to halt progression or prevent rupture is that there remains an unmet need in the knowledge gap of the causal mechanisms of AAA rupture. A second unfulfilled need in experimental pre-clinical models is treatment with pharmacologic therapies of well-established, chronic large AAAs to better resemble human disease. As more studies treat well-established AAAs and the knowledge gap closes, there will be more medical treatment therapies that better translate to human disease. The treatment of established AAAs with these untested inhibitors in the future could provide new knowledge of the function of NADPH oxidases in AAA formation and rupture and could provide potential treatment therapies for this deadly disease.

## 6. Conclusions

In summary, there is a growing body of evidence to suggest the importance of NADPH oxidases in AAA formation, progression, and rupture. Future studies investigating the roles of these enzymes in processes such as mitophagy, apoptosis, and senescence in aging in AAAs may provide new insight into the mechanisms of these enzymes in AAAs. Furthermore, investigation into mechanisms of known NADPH oxidase pharmacologic inhibitors could provide greater insights into the development of novel pharmacologic treatment therapies to halt AAA progression and to prevent AAA rupture. AAA rupture carries an approximate 50% morbidity and mortality rate, and there remains an unmet clinical need to provide medical treatment therapies to prevent AAA rupture. Perhaps targeting of NADPH oxidases in clinical applications could help halt AAA progression or prevent rupture.

## Figures and Tables

**Table 2:** Genetic elimination of NOX family members in experimental AAAs.

Gene Knockout	AAA Model	Influence on Experimental AAAs	References
NOX1	Angiotensin II	Decrease rupture rate, altered TIMP1 expression	[104]
	Angiotensin II	Decreased rupture, decreased size, decreased eNOS	[105]
NOX2	Angiotensin II	Decreased rupture, decreased size, decreased eNOS	[105]
	Elastase	Decreased size, decreased MMP2 and 9, decreased IL-17 and IL-23 production	[94]
NOX3	n/a	unknown	
NOX4	Angiotensin II	Decreased rupture, decreased size, decreased eNOS	[105]
	Fbn1^C1039G/+^	Decreased aneurysm, decreased elastin breakage	[108]
NOX5	Diabetic models + Nox5 overexpression	Increased aneurysms	[109]
DUOX1	n/a	Elevated but unknown	[110]
DUOX2	n/a	unknown	
p22^phox^	n/a	Elevated but unknown	[31,80,111]
p47^phox^	Angiotensin II	Decreased rupture, decreased MMP2	[112]
	Angiotensin II	Decreased rupture, decreased size, decreased eNOS	[105]
p67^phox^	n/a	Elevated but unknown	[80,111]
Rac1	n/a	Elevated but unknown	[80,111]
Rac2	n/a	Elevated but unknown	[113]

## Abbreviations

AAA	Abdominal aortic aneurysm
Ang II	Angiotensin II
ApoE	Apolipoprotein E
ASDR	Age-standardized death rates
eNOS	Endothelial nitric oxide synthase
HMGB1	High-mobility group box 1 protein
HIF-1α	Hypoxia inducible factor alpha
LDLR	Low-density lipoprotein receptor
NAPDH Oxidases	Nicotinamide adenine dinucleotide phosphate oxidases
PPARα	Peroxisome proliferator-activated receptor alpha
ROS	Reactive oxygen species
SMCs	Smooth muscle cells
